# Reducing violence against children by implementing the preventative intervention Interaction Competencies with Children for Teachers (ICC-T): study protocol for a cluster randomized controlled trial in Southwestern Uganda

**DOI:** 10.1186/s13063-018-2827-9

**Published:** 2018-08-13

**Authors:** Joseph Ssenyonga, Katharin Hermenau, Mabula Nkuba, Tobias Hecker

**Affiliations:** 1 Department of Psychology, University of Konstanz, 78567 Konstanz, Germany; 2 vivo international, 78340 Konstanz, Germany; 3 Department of Educational Psychology and Curriculum Studies, Dar es Salaam University College of Education, 2329 Dar es salaam, Tanzania; 4 Department of Psychology, Bielefeld University, Postbox 100131, 33501 Bielefeld, Germany

**Keywords:** School violence, Students, Behavioral problems, Secondary school, Teachers

## Abstract

**Background:**

An adolescent's school is often the second most important place for his development and education after the home. However, reports highlight the recurrence of the use of violent discipline in schools. There are few school-based interventions that aim at reducing violence at school that have been implemented and evaluated in sub-Saharan Africa. To reduce violent disciplinary measures used at school, we aim to implement and evaluate the feasibility and efficacy of the preventative intervention *Interaction Competencies with Children for Teachers (ICC-T)*.

**Methods/design:**

The study will be conducted in six randomly selected districts of the Ankole region in southwestern Uganda. We shall randomly select two mixed-day secondary schools from each district that fulfill our inclusion criteria. Schools will be randomly assigned to the intervention condition, where ICC-T will be implemented, and control schools (no intervention). Sixty students between the ages of 12 and 17 years and at least 15 teachers per school will be included in the trial. We aim to collect pre-assessment data directly before the intervention (t1) and 3 months after the intervention (t2) in both intervention and control schools.

Using self-administered questionnaires, we will measure students’ exposure to violence using the Conflict Tactics Scale (CTS), their psychological well-being using the Strengths and Difficulties Questionnaire (SDQ), and teachers’ positive attitudes towards violent disciplining and teachers’ use of violent disciplinary methods (CTS). The implementation feasibility of ICC-T in the cultural context of southwestern Uganda will be assessed with purpose-built measures that follow the guidelines for feasibility studies assessing the demand, applicability, acceptability, and integration of core elements in the daily work.

**Discussion:**

The proposed study will allow us to test the feasibility and efficacy of a preventative intervention seeking to reduce violent disciplinary measures in school settings using a scientifically rigorous design. The proposed study provides the opportunity to contribute to the attainment of goal number 16.2 of the United Nations’ Sustainable Development Agenda 2015–2030, which aspires to end all forms of violence against children.

**Trial registration:**

ClinicalTrials.gov, NCT03051854. Registered on 14 February 2017.

**Electronic supplementary material:**

The online version of this article (10.1186/s13063-018-2827-9) contains supplementary material, which is available to authorized users.

## Background

Violent disciplinary measures refer to the deliberate use of physical force that results in bodily and/or emotional pain with the aim of correcting or regulating a child’s behavior in the school setting [1]. Violent disciplinary measures are prevalent worldwide and take diverse forms, including the use of hands or objects, such as a cane, whip, or stick, by teachers and school staff to inflict bodily pain on the students [2]. Other forms of punishment include flogging, lashing, shaking, scratching, kicking, and pinching. Maintaining a seated position on an imaginary chair for long periods of time and adoption of bodily postures that cause enormous pain are other examples of methods of physical punishment used in the school setting [3]. In educational settings students can be exposed to violence, which may result in a variety of negative outcomes, including fear of the school staff, emotional problems, physical injuries, and mental health problems which, in the long term, may affect academic achievement [4].

### Global perspective on violence by teachers

Worldwide, the use of physical violence by teachers is legally accepted as a disciplinary measure in 68 countries [5]. Africa accounts for 40% of all countries globally which lawfully allow physical punishment in the education context. Students experience violence at school, especially in the USA and in Asian and African countries [3, 6, 7]. Providing global estimates of physical violence at school using data from 63 countries from Asia, Africa, Europe, and North and South America, one research report noted that the prevalence rates were between 13% and 97% among 29 countries with legislation that prohibits the use of corporal punishment in school, while 20 countries that do not prohibit violence by teachers in school had prevalence rates between 70% to 98%. Rates of physical violence at school were generally higher in low- and middle-income countries [3].

Despite the legal framework that prohibits physical violence at school, its use continues in many countries. This could be due, for example, to a lack of proper implementation of appropriate laws. Though many countries have enacted laws that forbid physical violence at school, they have failed to stipulate alternative forms of disciplinary procedures applicable in the school setting to guide teachers and students. This contradiction has resulted in teachers’ reliance and continued exclusive use of violent punishment as a disciplinary measure. Furthermore, there are many countries in which violent discipline is still legal [3, 8, 9].

### Violence by teachers in sub-Saharan African countries

Research reports have documented particularly high prevalence rates of violent punishment in sub-Saharan Africa. In total, 27 countries do not fully forbid physical and emotional violence by teachers, which increases the likelihood of students experiencing violence at school [3, 5]. Rates of violent punishment in 22 selected African countries (12 states that allow corporal punishment) range from 98% among boys and 91% of girls in Tanzania to 28% of students in Djibouti [3]. One study conducted among 42 primary schools in Ghana, Kenya, and Mozambique revealed that 80–90% of students experienced physical violence at school in the past year [10]. More than 52% of students experienced violence at school in West and Central African countries including Benin, Senegal, the Central African Republic, and Gambia [11]. Moreover, about half of in-school adolescents experienced physical violence in Namibian schools [12].

Violent disciplinary measures are used when students violate school norms, perform poorly, or make noise in class [3, 7]. Teachers justified the use of violence in the context of child discipline procedures and as a way of exercising power, compliance, and behavioral control [13]. Teachers preferred the use of corporal punishment because they perceived it to be an effective discipline measure that results in immediate compliance. In spite of their use of corporal punishment, teachers were not aware of the consequences associated with the use of violence and lacked knowledge of other effective discipline alternatives [14]. As a consequence, positive attitudes towards corporal punishment may result in the continuation of violence by teachers at school [13].

### Current situation in Ugandan schools

The use of violence at school in Uganda is prohibited by laws, policies, and guidelines, including the Teachers’ Professional Code of Conduct [15], the penal code of Uganda laws, the Education Act, and the Ministry of Education and Sports guidelines [16, 17]. Physical punishment was suspended in schools and colleges by 1997 and finally abolished in 2006 by the Uganda Ministry of Education and Sports; nevertheless, the use of corporal punishment still frequently occurs in the education setting to date [17].

One survey of 25 schools across five districts in Uganda found that 81% of the children had experienced physical violence at school [18]. More than 90% of primary school pupils had been exposed to physical and emotional violence at school [4]. Students experienced teacher-inflicted physical violence on an almost weekly basis [19]. These findings underline the fact that legislation has not resulted in the overall elimination of violence by teachers in Uganda.

### Consequences of violence by teachers at school

School violence is a painful experience associated with physical injuries [20, 21], child aggression and antisocial behavior [22], externalizing and internalizing behavioral problems [4], and depression and post-traumatic stress disorder (PTSD) symptoms [23–25]. Research findings also emphasize that students who have been victims of violence at school showed lower self-esteem, engaged in destructive avoidance behavior, and limited their communication strategies in the school locale [14]. In accepting violence as the normative disciplinary measure, students do not understand that the use of violent disciplinary measures is a violation of their rights [20].

Furthermore, the use of violence in the school leads to chronic fear of violent teachers as well as school avoidance [14]. Consistently, violence by teachers at school has resulted in negative education outcomes, such as low education achievement and school absence [21], poor academic performance, and increased school drop-out rates and absenteeism [18, 20].

### School-based violence prevention interventions

The use of physical violence is further reinforced by sociocultural norms that justify its use as a disciplinary method. Hence, there is a pressing need to reduce children’s exposure to violence, especially in the school setting [3]. This calls for prevention approaches against the use of violence by teachers at school.

Human rights activists at the global level have been at the forefront of advocating for the ban of corporal punishment [8]. For example, the United Nation’s Sustainable Development Goal Number 16.2 seeks to put an end to all kinds of violence against children by 2030 [26]. The African Union, in the same vein, seeks to protect children from violence. For example, the African Committee on the Rights and Welfare of Children envisaged that by the year 2020 countries in Africa ought to have outlawed the use of violence in the education sector and anticipate that by 2040 there will be no child who experiences violent disciplinary measures in any setting [27]. However, the focus so far has been mainly on the legislative aspect.

Thus, interventions that aim at preventing violence while changing attitudes and behavior in relation to violence become a necessity, particularly in contexts in which the use of violence is the norm rather than the exception [3]. Interventions that include training aspects for teachers seem to be successful in reducing violence at school. Recommended training content includes non-violent corrective approaches.

However, there are very few interventions, especially in low-income countries, that have been evaluated for their efficacy. For example, one study in South Africa examined the consistency between the disciplinary approaches used in the schools and the tenets of alternatives to corporal punishment. Generally, the implementation of alternative disciplinary strategies was hindered by the lack of formal training of educators in these methods and inadequate consultations with education stakeholders. Despite the noted challenges, alternatives to physical punishment resulted in better discipline among learners, provided teachers with more non-violent discipline choices, accorded students the opportunity to explain as much as possible for any noted behavioral deviations, and built a school culture based on self-discipline and non-violence [28].

In Uganda, the use of violent disciplinary approaches in schools is legally not permitted; however, teachers are not formally provided with alternative disciplinary strategies applicable in the education sector [15]. Positive disciplinary approaches [17] appropriate for schools in handling student discipline-related concerns have been proposed. These include reflection to tackle minor problems, penalties for persistent problems, suspension for offenses that cause damage to others and property, and as a last resort, suspension for consistently serious wrongdoings.

The Good Schools Toolkit intervention designed to prevent physical punishment against students has been evaluated in 42 Ugandan schools [29]. The kit engages stakeholders including teachers, students, parents, and school administrators in advocating for the use of non-violent discipline techniques as a way of fostering a better learning environment and mutual respect in schools among others. The study evaluated the implementation of the Good Schools Toolkit in primary schools in Luwero District in the Central Region of Uganda. In a cluster randomized controlled trial the Good Schools Toolkit intervention resulted in a significant reduction (42%) of teacher-initiated physical violence against students [21]. At follow-up, school staff reported using less violence in the past week at intervention schools (16%) than at control schools (33%; odds ratio 0.39, 95% confidence interval 0.20–0.73). The prevalence of past week physical violence reported by students was also lower in the intervention schools (31%) than in the control schools (49%; odds ratio 0.40, 95% confidence interval 0.26–0.64).

To the best of our knowledge, there is as yet no school-based violence prevention intervention that has been implemented and evaluated for its efficacy at the secondary school level in Uganda. The preventative intervention *Interaction Competencies with Children (ICC)* aims to foster better adult-child interactions while reducing the occurrence of violent discipline. There are currently two versions of ICC, one for caregivers (ICC-C) and one for teachers (ICC-T) [30–33]. ICC-T aims to contribute to the reduction of violence so that students do not experience emotional and physical violence at school in the long term [34]. In East Africa, ICC-T has been successfully implemented and evaluated for its feasibility and efficacy in a cluster randomized controlled trial in secondary schools [30] as well as for its feasibility at the primary school level in Tanzania [31]. The study at primary school level revealed that teachers who participated in the ICC-T program found the intervention content to be relevant for their work and were able to integrate the ICC-T tenets, such as alternative discipline methods, into their daily working routine. ICC-T resulted in better teacher-student relations, less physical violence by teachers, and improved student behavior in the follow-up assessment [31]. On the secondary school level, a cluster randomized controlled trial was implemented: eight schools were randomly assigned as intervention and control schools. Results showed that the participating teachers reported a high acceptance of the intervention and a good integration of ICC-T content into their daily work. At follow-up, there was a substantial difference in the use of both emotional and physical violence by the teachers as reported both by students (emotional violence: effect size Cohen's *d* = 0.94; physical violence: effect size partial η^2^ = .06 after controlling for difference at baseline) and teachers (emotional violence: *d* = 1.56; physical violence: *d* = 1.38). Teachers’ positive attitudes towards violence were also lower in the intervention schools at follow-up (emotional violence: *d* = 1.17; physical violence: *d* = 0.96) [30].

Encouraged by these promising initial results, in our current study we aim to evaluate the feasibility and efficacy of ICC-T at the secondary school level in southwestern Uganda. The implementation of ICC-T in Uganda builds upon previous knowledge, because ICC-T focuses on both physical violence and also emotional violence—the most common types of violence that co-occur in the school setting in Uganda [18, 19]. Moreover, during the interactive training phase, the views and needs of the teachers are incorporated into the training.

### Objectives

The use of violent discipline strategies has continued in Ugandan schools to date despite guidelines that hinder the use of punitive correction approaches. This has been complicated by strong cultural beliefs and support for the use of violence in schools and the general lack of formal alternatives to physical punishment that can help teachers to handle student discipline concerns. To address this challenge, we will implement and evaluate the feasibility and efficacy of ICC-T as a violence prevention approach in secondary schools in southwestern Uganda. With ICC-T our goals are to change teachers’ attitudes concerning the use of violent disciplinary measures, reduce the use of harsh and violent disciplinary measures in schools, and foster better interactions between students and teachers.

## Methods/design

### Study design

**Fig. 1:**
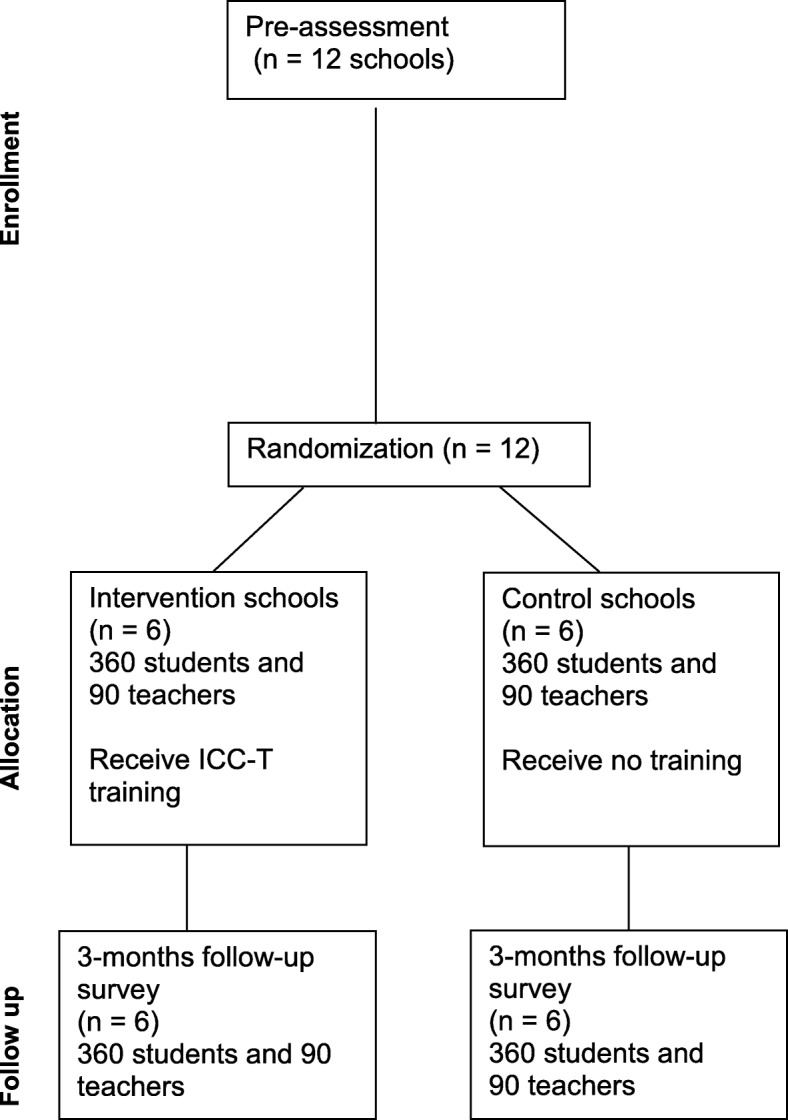
Flow chart of the study design

**Fig. 2:**
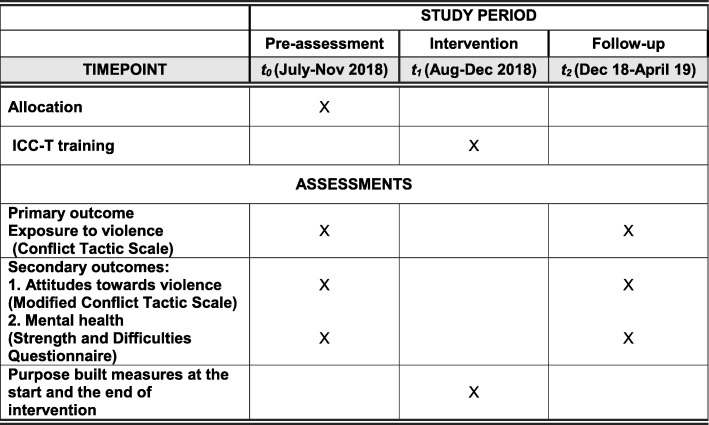
Participant timeline chart

In a two-arm cluster randomized controlled trial, 12 secondary schools will be randomly assigned to the intervention group (which will receive the ICC-T intervention training) or the control group (which will receive no training). The study will have two data collection points: pre-assessment and follow-up assessment. See Figs. 1 and 2 and the Standard Protocol Items: Recommendations for Interventional Trials (SPIRIT) checklist (Additional file 1).

### Study setting

In Uganda, primary school takes 7 years, secondary school (ordinary level) 4 years, and high school (advanced level) 2 years. The education sector in Uganda is divided into 13 regional clusters, with each cluster having seven to 15 districts. Southwestern Uganda has two regional blocks: Ankole and Kigezi. This study will be conducted in Ankole region. Ankole has the third highest student enrollment in Uganda with 134,509 students (50% males), and the majority of the secondary school students (47%; *n* = 62,807) studying in *Senior One* and *Senior Two* (8th and 9th years of formal schooling). The region has 10 districts with each district having 3–20 government-aided secondary schools.

Ankole region was purposefully selected because it has the second largest number of government-aided secondary schools (117), with 96 of those implementing the free Universal Secondary Education (USE) government program. Government-aided schools implementing the USE program as a matter of policy are expected to have at least two streams with 60 students per stream. This translates to a minimum secondary school student population of 720 students [35].

The Ministry of Education, Science, Technology and Sports reports that there were only 80 mixed secondary schools that implemented the USE program in the region, which included the number of classes, school enrollment, and number of teachers [35, 36]. Ankole region has 3460 teachers employed in the various schools. The 117 government-aided secondary schools in the region have on average about 25 teachers [36].

### Schools

We plan to include 60 students (30 in 8th year and 30 in 9th year of formal schooling) per school. Based on previous studies in similar settings, we expect a participation rate of approximately 50% [23, 37–39]. This means that we will include schools that have a minimum student enrollment of 60 per year of study, i.e., 360 students in total.

Furthermore, as ICC-T intervention is a participatory approach that involves active engagement and practical hands-on learning, we consider ICC-T workshops to be cost-effective with a minimum number of at least 15 participants. That is why we will include only schools that employ at least 15 teachers. Only 41 schools fulfilled these inclusion criteria.

Additionally, we aim to select at least two schools per district in order to randomly assign them into the intervention or control conditions. Eight districts, i.e., Bushenyi, Ibanda, Isingiro, Kiruhura, Mitooma, Ntungamo, Mbarara, and Sheema, have at least two eligible schools. Given that Mbarara district is more like the “regional capital” of the Southwestern region, we purposefully included this district in the study sample. We then randomly selected five districts from the remaining seven districts. In the event that the selected district has two eligible schools, these schools are automatically included in the study sample. From districts that had three to 11 eligible schools, only two schools are randomly selected. As a result, the final sample consists of 12 coeducational secondary schools, from six different districts: Ibanda, Isingiro, Kiruhura, Mitooma, Ntungamo, and Mbarara.

### Participants

Due to the longitudinal nature of the study which necessitates two data collection points, the focus of this study is on students in the 8th and 9th years of formal schooling. Based on a previous study that used a similar design to test the efficacy of ICC-T in Tanzania [30], we would expect a moderate to large effect on students’ self-reported exposure to violence. An a priori power analysis (α = .05, power = 0.80, moderate effect size of ƒ = 0.25) using G*Power software [40] indicated a required total sample size of at least *n* = 128 students to detect significant interaction effects. To adjust for the nested design of the study, we calculated the following design effect (DE): DE = 1 + (fixed cluster size considering drop-outs − 1) × intra-cluster correlation coefficient. Considering 60 students per school, a drop-out rate of 20%, and an intra-cluster correlation coefficient of 0.05, the DE for the student sample is 3.35, which results in a required sample size of at least 430 students. We aim to randomly select, at each school, 30 students from Senior One (8th year) and 30 from Senior Two (9th year). The target sample will thus be 720 students in the age range between 12 and 17 years. At the classroom level a list of all students will be obtained from the school administration. Stratified random sampling will be used to select 15 boys and 15 girls from each class or stream.

All teachers who are officially working in the selected schools will be included in the study sample. Based on the previous study in Tanzania [30], we would expect a large effect on teachers’ self-reported use of violence against students. An a priori power analysis (α = .05, power = 0.80, moderate to large effect size of ƒ = 0.35) using G*Power software [40] indicated a required total sample size of at least *n* = 67 teachers to detect significant interaction effects. To adjust for the nested design of the study, we again calculated the DE. Considering a minimum of 15 teachers per school, a drop-out rate of 20%, and an intra-cluster correlation coefficient of 0.05, the DE for the teacher sample is 1.55, which results in a required sample size of at least 104 teachers. Our target sample will be at least 15 teachers per school, resulting in a total sample of at least 180 teachers.

Only selected students aged 12–17 years and teachers in the age range 18–65 years, employed by the selected schools and who will be present during the data collection period, will be included in the study. Teachers will be enrolled in the study if they give their informed consent. Students with parental informed consent and who freely provide assent will be accepted to participate in the study. Students and teachers with acute psychotic symptoms or acute alcohol or drug intoxication will be excluded from the study.

### Procedure

One member of the study team has already visited the selected schools and informally discussed the research with the school administrators. All the selected schools agreed to participate in the study, and the school head teachers provided support for the study. During the subsequent visit to the schools, formal study introduction letters and supporting documents will be provided to the school administrators. The research team members will then explain pertinent study details to the school authorities.

Informed consent will be sought from the teachers before they participate in the study. The relevant details pertaining to the study, including the purpose and significance of the study and ethical concerns including privacy, confidentiality, legal rights, and informed consent, etc., will be explained in detail to the selected participants in English. The research team will also respond to any identified participants’ concerns in relation to the proposed study.

Informed consent from parents and assent from students will be obtained before the students are enrolled in the study. Selected students will be given a parental consent document that they will take home to their parents to sign or thumb-print. The consent document written in English and Runyankole will provide the parents with relevant information about the study including the ethics involved in the study. After obtaining parental consent, the students will assent before they are formally enrolled in the study.

Students in Uganda use English as the medium of instruction throughout the educational cycle. Therefore, the study questionnaire will be administered in English. A pilot study was conducted at one coeducational secondary school in Mbarara district. The pilot test verified the practicality of the data collection procedure and assessed the ease of use of the research instrument.

During the data collection period, the research team will closely supervise the students as they fill in the questionnaire. Research team members will each administer and supervise small groups of about three to five students each as they complete the questionnaire. Previous studies in Africa indicated that children and adolescents provide accurate and reliable information during research [21, 23, 32].

A set of questionnaires will be administered to the teachers, too. The research team will be available during the data collection period and will oversee the completion of the questionnaire. Additionally, in case of any clarification or request for more information, the research team will be available to attend to any concerns raised by the selected study participants.

### Intervention

ICC-T intervention is a training workshop, which lasts for 5.5 days, for teachers, with 8 hours spent in training on each full day. ICC-T aims at improving teacher-student relationships, changing teachers’ attitudes and behaviors concerning the use of violent disciplinary measures, and preventing harsh and violent discipline in the school setting. The ICC-T core ideas are based on the childcare guidelines of the American Academy of Pediatrics [41].

ICC-T follows core tenets, including incorporating a participative method in which the teachers are encouraged to take an active role during the workshop. Theory and practice are combined during the workshops to enable the teachers to integrate the attained ICC-T skills into the daily work routine at school. During the workshop, confidentiality is emphasized to enable the teachers to freely speak about their work-related tribulations, their desires, and experiences with violent discipline in a trusting and welcoming environment. ICC-T’s sustainability is achieved through rigorous rehearsal of previously learned material, teambuilding events, support supervision, peer consultation, formation of referral networks, and personal reflection on personal behavior. ICC-T ensures that the acquired skills and knowledge will be integrated into the teacher’s everyday school endeavors. Likewise, sustainability is achieved through provision of feedback during the course of the training and through case discussions.

ICC-T is based on five essential components that foster better student-teacher relations and a reduction in school physical punishment. ICC-T training has sessions on teacher-student interactions, maltreatment prevention, effective discipline strategies, identifying and supporting burdened students, and practical implementation of ICC-T aspects in the school setting.

Sessions on teacher-student interactions include topics such as communication skills, instructions and expectations, teachers as role-models, and rules in the classroom. These sessions assist teachers in understanding students’ behavior and highlight teachers’ responsibility as role models for the students. The sessions aim at improving teacher-student relations.

Maltreatment prevention sessions discuss the undesirable outcomes of violent disciplinary measures. Teachers use self-reflection to make a connection between their own childhood experiences of violent punishment, their current use of violent punishment, and its consequences. Discussion topics in this session consist of frequent disciplinary methods, myths about the utility of violent punishment, consequences of violent disciplinary methods, and alternative discipline approaches.

Session on effective discipline strategies intend to equip teachers with non-violent alternatives. Through role-plays teachers will learn how to use non-violent strategies practically, such as privilege removal and reinforcement, to foster desired behavior.

Teachers need to effectively recognize and assist troubled students. This task requires teachers to acknowledge that students may suffer from emotional and behavioral problems. This session will discuss the common internalizing and externalizing behavior problems, developmental delays, and student stress. Afterwards, diverse methods of assisting distressed students will be discussed.

Sessions on ICC-T implementation aim for integrating the learned material into the daily work routine in the school setting. Successful implementation includes collaboration with school staff and peer consultation.

The proposed training strategies include presentations, discussions, question and answer sessions, and supervised practical sessions. The training will be based on the previous success of ICC-T intervention training for teachers in Tanzania. The training was feasible, and first evidence of its effectiveness was found, e.g., a change in teachers’ positive attitudes towards emotional and physical violence and the use of and exposure to physical and emotional violence reported by students and teachers [30, 31].

### Control

At the randomly selected control schools no intervention will be implemented. However, the study will control for the potential influence of other workshops for teachers that may take place during the course of the study. The school administrations will provide information on all such programs during the course of the study.

### Outcome measures

Our study intends to test the effects of ICC-T training on the use of violence by teachers at school. This primary outcome measure is assessed by students’ self-reported experiences of violence (emotional and physical violence) as well as teachers’ self-reported use of violence (emotional and physical violence). Secondary outcome measures include teachers’ attitudes towards violence (emotional and physical violence) as well as student’s mental health (see Fig. 2).

All measures selected for the trial have been used in previous studies in East Africa. Further, the reliability coefficients of the instruments in those studies were acceptable [21, 23–25, 30–32, 37, 38].

#### Students

##### Exposure to physical and emotional violence

The Conflict Tactics Scale (CTS) will assess exposure to common disciplinary measures at school from the students’ perspective. The original CTS assesses diverse disciplinary behaviors including physical assault, emotional and psychological aggression, neglect, and non-violent discipline. For the current trial a modified version of the CTS [30, 37, 38] that has been used in previous studies in Tanzania will be implemented. It measures physical violence with 13 items and emotional violence with five items. Items are rated on a 7-point Likert scale from “never” scored as 0 to “more than 20 times” scored as 6. Subscale scores are derived by summing up the item scores. Physical violence scores range from 0 to 78, while the emotional violence scores range from 0 to 30. The scale has acceptable internal consistency for physical violence (α = .55) and emotional violence (α = .69) in previous studies. The scale reliabilities are acceptable, as the scales assess situations where the items measure relatively exceptional incidents [1].

##### Mental health problems

The Strengths and Difficulties Questionnaire (SDQ) will measure the students’ behavioral problems, namely internalizing and externalizing problems. The 25-item SDQ assesses four problem behaviors: emotional problems, peer problems, conduct problems, and hyperactivity. Each subscale has five items rated on a scale ranging from “not true” (0) to “certainly true” (2). Reversed items are recorded before the computation of the total scale score (sum of scores for hyperactivity, emotional symptoms, conduct problems, and peer problems) that ranges between 0 and 40, with a score above 20 representing the presence of mental health problems. The Cronbach alpha reliability of the total difficult score was .82 [42]. Internalizing problems including peer problems and emotional symptoms had Cronbach’s alpha coefficients of .61 and .75, respectively. Internal reliabilities for externalizing problems were .72 for conduct problems and .69 for hyperactivity in the pilot validation study [42].

#### Teachers

##### Purpose-built measures for ICC-T training evaluation

The purpose-built measures adapted from Kaltenbach et al. [31] and Nkuba et al. [30] will be used to assess the feasibility of ICC-T in the cultural context of southwestern Uganda. We follow the guidelines for feasibility studies by Bowen et al. [43] in assessing the demand, applicability, acceptability, and integration of ICC-T core elements in the teachers’ daily work. The demands will be assessed through examination of teachers’ positive attitudes towards violent disciplining before and directly after training. The applicability of the training (e.g., expectations about the workshop, relevance of the workshop) will be measured before the intervention, directly after the intervention, and at the follow-up assessment. Furthermore, we will examine the acceptability of the training (e.g., satisfaction with the training, evaluation of new knowledge) directly after the intervention and at the follow-up assessment. Finally, we will assess the integration of the ICC-T core elements in teachers’ daily work at school directly after the intervention and at the follow-up assessment.

As measures of efficacy we will assess the change in attitudes towards and use of violent disciplinary measures as well as a perceived change in the teacher-student relations (e.g., How did the training influence your understanding of students?). After a 3 months follow-up, integration and implementation of learned ICC-T material into daily work routines will be measured. Attitudes towards emotional and physical violence and actual application of violent discipline strategies in the school will be assessed using items from the modified CTS that have been used previously in Tanzanian studies [30, 31, 37, 38]. Teachers will respond to the 18 items (13 items measure physical violence, and 5 items measure emotional violence) of the CTS with regard to the use of school violence. The CTS items are scored using a 7-point answer category from “never” scored as 0 to “more than 20 times” scored as 6. Subscale scores are derived by summing up the item scores. Physical violence scores range from 0 to 78, while the emotional violence scores range from 0 to 30.

Moreover, teachers will be asked to report their attitudes towards violent discipline strategies using the 18-item modified CTS. The items are scored on a 4-point Likert scale from “never OK” scored as 0 to “always or almost always OK” scored as 4. Subscale items are summed up to yield scores for physical violence (range 0–52) and emotional violence (range 0–20).

### Analysis

Primary analysis will be carried out based upon the groups as randomized (“intention to treat”). We will use the last-observation-carried-forward approach; i.e., in drop-outs we assume no change from pre-assessment to follow-up. Results will be presented including appropriate effects sizes and a measure of precision (95% confidence intervals).

Our main analysis of the primary outcomes, students’ exposure to and teachers’ use of physical and emotional violence, will be *time × group* interaction effects using repeated multivariate analysis of variance (MANOVA). In case of a noted cluster effect (intra-cluster correlation coefficient > .10) we shall use multilevel analysis. Multivariate interaction effects and the univariate interaction effect of each outcome variable will be tested first. Paired *t* test analysis will examine the differences from the pre-assessment to follow-up assessment in the intervention group while the independent *t* test will examine whether there is a difference between the control group and intervention group at the follow-up assessment. Effect sizes η^2^ ≥ 0.01, η^2^ ≥ .0.06, and η^2^ ≥ 0.14 will be considered to represent small, moderate, and large effect sizes correspondingly. For *t* tests effect size interpretation will be guided by the suggestion of Cohen where *d* ≥ 0.20, *d* ≥ 0.50, and *d* ≥ 0.80 will represent small, medium, and large effect sizes, respectively.

### Ethical considerations

Given that the research involves human subjects considered as a vulnerable group, i.e., children [44], ethical clearance was obtained from the relevant ethical boards. The Mbarara University of Science and Technology Research Ethics Committee (MUST15/10-15), the Uganda National Council of Science and Technology (SS 4032), and the University of Konstanz Ethic Review Board (35/2016) have already approved the study.

Only pre-assigned codes will appear on the questionnaires and consent documents. Data will be stored on a password secured computer accessible to only the study investigators. Data obtained during the research will be kept confidential and will not be disclosed to another person without the participant’s permission or as required by the law. Behavioral intervention studies are minimum risk studies. However, in case of any unexpected adverse effect the researchers will document and report such occurrences to the respective ethical bodies within 1 week. Questions about experiences may evoke upsetting memories in the event that the participant experienced similar events in his or her life. Participants who will experience any psychological distress during the course of the data collection will be provided with psychological support by the research team members. For participants who experience adverse or unexpected events, appropriate referrals and follow-up for specialized services and further management will be made on a case-by-case basis.

## Discussion

Research findings, media reports, and non-governmental organization reports have provided anecdotal evidence about the prevalence, magnitude, and consequences of violence against children in Uganda [8, 13, 17–19, 45]. However, violence in school settings is still prevalent in Uganda despite efforts to protect children from violence through legal means. While Uganda has policies that ban violence by teachers at school [15, 16], these legal measures need to be followed up with practical ways of handling disciplinary issues in the education locale. Despite the increase of violence against children, few school-based interventions which aim at reducing violence by teachers have been evaluated for their effectiveness [3]. In Uganda, no violence prevention interventions have been scientifically evaluated at the secondary school level to the best of our knowledge.

Our study aim is to implement and evaluate the ICC-T intervention, which aims to reduce violence by teachers at school. The study will adopt a two-arm cluster randomized controlled trial design, with six schools allocated to the intervention group and six control group schools. The study will use a large sample that is representative for the government-aided secondary schools in the southwestern region of Uganda. The experimental design will allow referring of potential interaction effects to the intervention, and it controls for most potential confounds. Additionally, our results may be generalizable to similar school settings in Uganda and the region.

The study takes on a multi-informant approach, as data will be collected from a sample of teachers and students. We shall elicit self-reports from students and teachers in relation to school violence. Hence, the teachers’ self-reports will, in part, be complemented by the viewpoints of students. Furthermore, the instruments adopted for the study have a good theoretical basis and have proven to be reliable in measuring students’ exposure to and teachers’ use of school violence and in screening for mental health problems in East Africa [1, 21, 30, 32, 37, 38, 42].

The proposed study results may have implications for schools, teacher training, and policy in Uganda. Teachers’ continued use of violence despite policies that ban its use points to problems with teacher training in Uganda. Intervention approaches, such as ICC-T, that reduce violent disciplinary measures need to be implemented and evaluated at the school level. This may result in the frequent use of non-violent disciplinary methods in schools. Furthermore, regular teacher training needs to be enriched with respect to management of students’ behavior, alternatives to corporal punishment, and fostering of better teacher-student relations. Thus, the results of our study may help the Ministry of Education, Science, Technology and Sports to implement the relevant guidelines and programs that prohibit school violence in a more practical way. Moreover, there is a need to inform education sector stakeholders about the laws that ban the application of violent disciplinary measures in schools, children’s rights, teacher code of conduct, domestic violence laws, and the consequences of violating the applicable laws. Reporting procedures, monitoring guidelines, and review mechanisms must be enshrined in the broader legal implementation plan [8, 15, 16]. Research findings — to which our proposed study may also contribute — are vital in helping the population at large understand the negative effects of school violence.

The proposed study has some limitations. Self-report questionnaires are prone to possible respondent bias and social desirability. Furthermore, the proposed 3-month period between the intervention and follow-up assessment is rather short. The anticipated changes in attitudes and behavior can be regarded as preliminary in nature. Further, the inclusion of relatively few schools limits the generalizability of the study findings. One anticipated problem is the fluctuations in the number of respondents. Teacher attrition can be associated with retirement, routine transfers of civil servants, and resignation, among other causes, while students can drop out of school or transfer to other schools not in the study area. Additionally, there are strong sociocultural factors, attitudes, and beliefs that support the use of violence against children. Nevertheless, involving the teachers in creating the change and formulating their own training may help to promote engagement in the process. Reflections about the teachers’ own experiences of harsh punishment and violent discipline, discussions about consequences of violence for children, and the intensive practice of effective non-violent discipline strategies may facilitate a change of attitude regarding violent discipline. We thus believe that the intervention may enable teachers to visualize the link between violence and the associated negative consequences. This may persuade teachers to embrace alternative disciplinary approaches in schools.

ICC-T is an interactive intervention in which teachers can learn how non-violent discipline measures can be implemented in a real-life school setting in a practical way. It is easily applicable to the school settings in low-income countries and can be scaled up to other government-aided schools in Uganda. Making the school environment a safe place that is free from violence has great potential to contribute to the attainment of Goal Number 16.2 of the United Nations Sustainable Development Goals 2015–2030, which aspires to end all forms of violence against children.

### Trial status

The trial preparation phase is ongoing until July 2018. Intervention pilot test took place in May 2018. Pre-assessment (control and intervention schools) is scheduled from July–November 2018. Interventions are planned from August until December 2018. The follow-up phase will start in December 2018 and end by April 2019.

## Additional file

Additional file 1: SPIRIT 2013 checklist: Recommended items to address in a clinical trial protocol and related documents. (DOC 120 kb)

## Abbreviations

CTS: Conflict Tactics Scale
DE: Design effect
ICC: Interaction Competencies with Children
ICC-C: Interaction Competencies with Children for Caregivers
ICC-T: Interaction Competencies with Children for Teachers
MANOVA: Multivariate analysis of variance
SDQ: Strengths and Difficulties Questionnaire
USE: Universal Secondary Education
